# Data on migration of the non-invasive breast cancer cell line, MCF-7 treated with Bevacizumab using Real Time Cell Analyzer (RTCA)

**DOI:** 10.1016/j.dib.2018.12.059

**Published:** 2018-12-21

**Authors:** Layal EL-Hajjar, Abdullah Shaito, Nour Jalaleddine, Kazem Zibara, Jalal M. Kazan, Jamal El-Saghir, Marwan El-Sabban

**Affiliations:** a Department of Biological and Environmental Sciences, Faculty of Science, Beirut Arab University, Beirut, Lebanon; b Department of Biological and Chemical Sciences, Faculty of Arts and Sciences, Lebanese International University, Beirut, Lebanon; c ER045 – Laboratory of Stem Cells, PRASE, Biology Department, Faculty of Sciences, Lebanese University, Beirut, Lebanon; d Department of Anatomy, Cell Biology and Physiological Sciences, Faculty of Medicine, American University of Beirut, Beirut, Lebanon

**Keywords:** Bevacizumab, MCF-7, Migration, RTCA

## Abstract

Bevacizumab or Avastin® (Av), the recombinant antibody targeting VEGF, improves progression-free but not overall survival of metastatic breast cancer patients due to development of Av resistance. We showed that Av-therapy-induced inflammatory microenvironment contributes to the refractoriness to Av treatment. Here we present data regarding the effect of Av treatment on migration of a non-invasive breast cancer cell line, MCF-7. The data presented hereis related to the research article “Bevacizumab induces inflammation in MDA-MB-231 breast cancer cell line and in a mouse model” (Hajjar et al., 2018).

**Specifications table**

**Table:** 

Subject area	Biology
More specific subject area	Cell Biology
Type of data	Image, Graph
How data was acquired	Real Time Cell Analyzer (RTCA) and quantitative PCR (qPCR)
Data format	Analyzed
Experimental factors	Samples were treated with Bevacizumab
Experimental features	Cell Proliferation and migration was performed using Real Time Cell Analyzer and gene expression analysis was done by qPCR
Data source location	Lebanon
Data accessibility	Data is with this article.
Related research article	Layal EL-Hajjar, Nour Jalaleddine, Abdullah Shaito, Kazem Zibara, Jalal Kazan, Jamal El-Saghir and Marwan El-Sabban. “Bevacizumab induces inflammation in MDA-MB-231 breast cancer cell line and in a mouse model”. Available online. https://doi.org/10.1016/j.cellsig.2018.11.007[1]

**Value of the data**

• The data reveals the motility behavior of Av-treated MCF-7 cells which may be interesting for researchers studying Av on non-invasive cell lines.
• The data may be relevant for other researchers investigating the potential of this treatment to promote growth and metastasis of cancer cells.
• The data may be important for researchers working on the effect of Av on non-transformed breast epithelial cells.
• The data provides the basis for *in vivo* and clinical studies.

## 1 Data

**Fig. 1:**
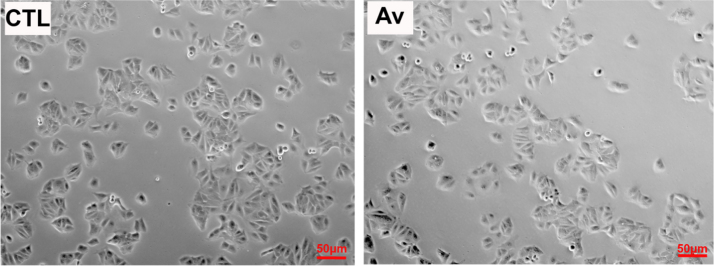
Av treatment showed no effect on cell morphology of Av-treated cells.

**Fig. 2:**
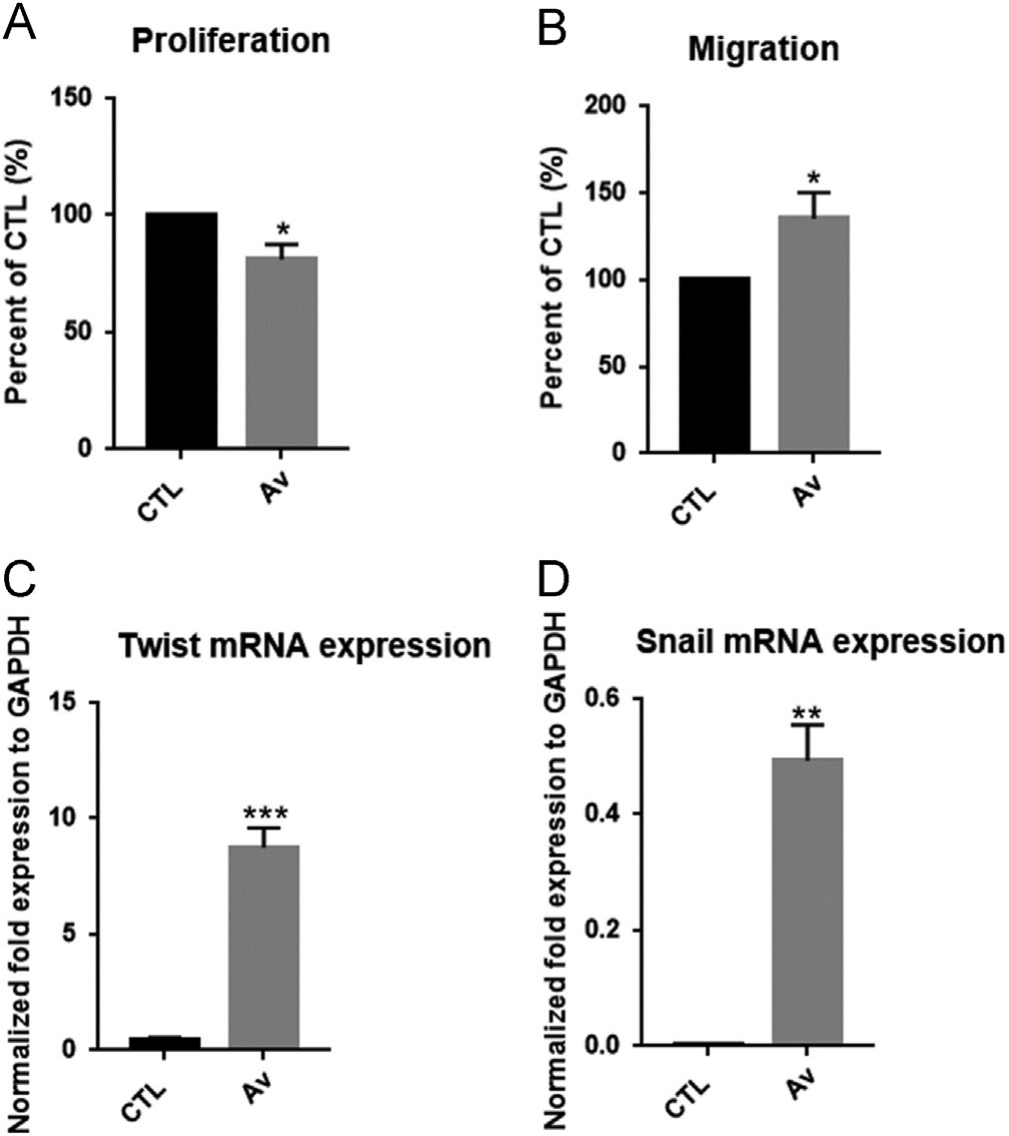
Av treatment affects proliferation and migration of MCF-7 cells as detected by RTCA and induces the expression of EMT markers as determined by qPCR. (A) Histograms representing the proliferation and (B) migration of Av-treated cells, after normalizing cell index values relative to controls. Cell impedance readings were recorded every 15 min for a minimum of 18 h. Histograms of Twist (C) and Snail (D) expression in Av-treated cells as detected by qPCR. Results represent three independent experiments. *, **, *** indicate *P* < 0.05, *P* < 0.001, *P* < 0.0001; respectively.

MCF-7 cells were treated with 50 μg/ml of Av for 24 h. MCF-7 cells showed no change in their morphology (Fig. 1). RTCA was used to measure the proliferation and migration of treated MCF-7 cells. The interaction of cells with a gold electrode correlated with impedance, which was reported as the cell index. Cells were treated, trypsinized and seeded in RTCA E- and CIM- plates to assess proliferation and migration, respectively. An inhibition of cellular proliferation by 22% in MCF-7cells was observed. There was an increase in migration which reveals the motility behavior of Av-treated MCF-7 cells. This observation was accompanied by a significant increase in mRNA expression levels of epithelial to mesenchymal transition (EMT) markers (Twist and Snail) (Fig. 2).

## 2 Experimental design, materials and methods

### 2.1 Migration and proliferation Real Time Cell Analyzer (RTCA) assays

Quantitative analysis of the effect of Av treatment on the proliferation and migration of MCF-7 cells was performed as previously described [2] with slight modifications using RTCA(×CELLigence RTCA[A2]DP, Roche Applied Science, USA). Cells were grown in 6-well tissue culture plates at a density of 10,000 cells/cm^2^ and treated or not with 50 μg/ml Av for 24 h. For migration assays cells were harvested, counted, re-suspended in 120 μl of serum-free media and seeded at a density of 20,000 cells/well in the upper chamber of CIM-plates. For proliferation assays, cells were seeded similarly, but in an E-plate at a density of 7000 cells/well with an additional 120 μl of media containing 10% serum. Migration and proliferation were monitored every 15 min for a minimum of 18 h by recording the cell impedance produced as the cells attached and detached from the gold electrodes in the CIM and E-plates. The RTCA software generated a survival curve and estimated the cell survival or cell index (CI). CI correlates directly with cell number. Data were expressed as bar graphs of CI % of control.

### 2.2 RNA extraction and qPCR

Total RNA was isolated from cells in culture using Nucleospin® RNA II Kit (Machery-Nagel, USA) according to the manufacturers’ instructions. 1 μg of total RNA was first reverse transcribed to cDNA using RevertAid 1st strand cDNA synthesis kit (Thermo, USA) and then amplified by qPCR using iQ SYBR Green Supermix in a CFX96 system (Bio-Rad Laboratories, USA). Primers were designed against human genes (TIB MOL BIOL, Germany) with the following sequences:

Twist: F: AGCTACGCCTTCTCGGTCT and R: CCTTCTCTGGAAACAATGACATC
Snail: F: CTTCCAGCAGCCCTACGAC and R: CGGTGGGGTTGAGGATCT
GAPDH: F: TGGTGCTCAGTGTAGCCCAG and R: GGACCTGACCTGCCGTCTAG

ΔΔCq was used to calculate the relative fold change in gene expression after normalization to the housekeeping gene, GAPDH.

### 2.3 Statistical analysis

Results are expressed as average ±SEM. Statistical comparisons were done using student׳s t-test in order to determine statistical significance. *P* value was determined and significance level was set at *P* < 0.05. Microsoft Excel was used to perform statistical analysis.
