# Supplementary material for: Data on migration of the non-invasive breast cancer cell line, MCF-7 treated with Bevacizumab using Real Time Cell Analyzer (RTCA)
Source: Data Brief. 2018 Dec 21;22:635–8. doi: 10.1016/j.dib.2018.12.059 (PMC6327074; doi:10.1016/j.dib.2018.12.059)
Supplement: Supplementary file 1 — Transparency document [file mmc1.docx]

**Conflicts of interest**

The authors declare no competing financial interests.
